# Tableau of the Yellow Fever of 1853, Etc.

**Published:** 1854-08

**Authors:** 


*Tableau of the Yellow Fever of* 1853, *with Topographical, Chronological, and Historical Sketches of the Epidemics in New Orleans since their origin in* 1796; *illustrative of the Quarantine Question.* By BENNET DOWLER, M.D. New Orleans. 1854.

THIS is a graphic little work, of 66 pages, and embraces apparently all that is known of the history of yellow fever in New Orleans for the last fifty-eight years. We have neither time nor space to do more than assure our readers that it is marked by the industry and ability characterizing all the productions of its distinguished author. It is truly a valuable acquisition to the literature of epidemics. J.

